# Age, sex, and vessel region affect the vasomotor function and gene expression signature of the aorta in mice

**DOI:** 10.1016/j.jmccpl.2025.100491

**Published:** 2025-10-19

**Authors:** Lars Saemann, Lotta Hartrumpf, Adrian-Iustin Georgevici, Sabine Pohl, Anne Großkopf, Kristin Wächter, Yuxing Guo, Andreas Simm, Gábor Szabó

**Affiliations:** a Department of Cardiac Surgery, University Hospital Halle, Martin-Luther University Halle-Wittenberg, 06120, Halle (Saale), Germany; b Department of Anaesthesiology, St. Josef Hospital, Ruhr-University Bochum, 44791, Bochum, Germany; c Department of Cardiac Surgery, University Hospital Heidelberg, 69120, Heidelberg, Germany

**Keywords:** Aging, Vascular aging, Gender medicine, Endothelium, Senescence, Endothelial integrity

## Abstract

**Introduction:**

Vascular aging is associated with endothelial dysfunction, changes in vascular elasticity or stiffness, and the prevalence of cardiovascular diseases. Aging differs by sex. The effects of age, sex, and vessel region on arterial vasomotor function and gene expression signatures have not been explored yet. Thus, we investigated contraction, relaxation, and endothelial integrity, as well as gene expression, in the proximal and distal segments of the thoracic aorta in 6- and 18-month-old mice.

**Materials and methods:**

Male and female C57BL/6J mice at 6 (*n* = 11/sex) and 18 (*n* = 12/sex) months of age were used. Segments of the proximal and distal thoracic aorta were mounted in organ bath chambers. We assessed the maximal receptor-independent contractility using potassium chloride (KCl), endothelial integrity using phenylephrine (PE), endothelial-dependent relaxation using acetylcholine (ACh), and endothelial-independent relaxation using sodium nitroprusside (SNP). Using microarrays, we performed transcriptomics on another 6 six mice of every subgroup.

**Results:**

Endothelial integrity decreases significantly with age in male mice, but only in the proximal segment. The relaxation to ACh decreases with age in both sexes in the proximal and only in female individuals in the descending segment. In females, endothelial-dependent relaxation is higher than in males, in young age, independent of the segment, and in old age, still in the proximal segment. Endothelial-independent relaxation decreases with age only in the distal segment of female subjects. Genes associated with the electron transport chain, crucial for energy production in mitochondria, are decreased by age. The G-protein coupled receptor -G13 alpha subunit- signaling pathway and proteasome degradation, which are crucial for developing and maintaining endothelial integrity, were reduced in the aorta of old mice. Genes involved in endothelial nitric oxide synthesis were especially downregulated in old male mice.

**Conclusion:**

Endothelial integrity and endothelial-dependent relaxation depend on age, sex, and segment of the descending thoracic aorta in mice. Genes associated with endothelial-dependent relaxation, endothelial integrity, and vascular aging change markedly by age, including some sex- and segment-specific differences.

## Highlights

• Endothelial integrity and endothelial-dependent relaxation depend on sex and segment of the aorta and deteriorate with age.
• The endothelial integrity deteriorates with age, most in the proximal segment of the descending aorta of male mice.
• Female sex is associated with high endothelial-dependent relaxation at a young age, independent of the segment.
• The expression of genes associated with endothelial relaxation changes markedly by age, including sex-specific differences.

## Graphical abstract

**Figure:**
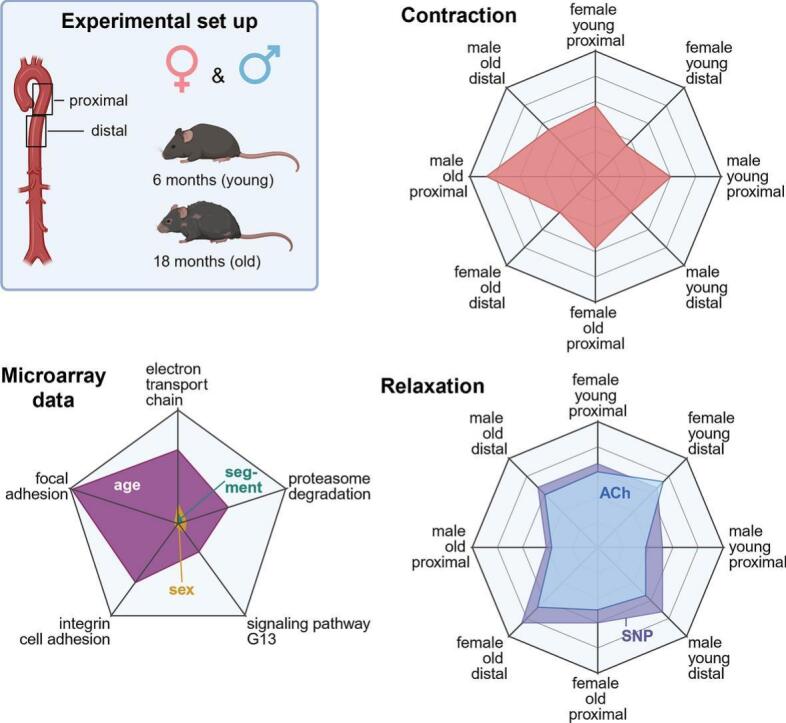

## 1 Introduction

Cardiovascular diseases are the leading cause of death [1]. Aging plays a significant role in the development of cardiovascular diseases [1]. It is known that vascular aging is associated with endothelial dysfunction, changes in vascular elasticity or stiffness, and the prevalence of arteriosclerosis [2]. An increased vascular tone accompanied by endothelial dysfunction promotes the development of arterial hypertension, a highly prevalent disease in the elderly [3]. Aging also contributes to the development of thoracic aortic aneurysms, although the exact mechanisms are not fully understood [4,5]. It has been shown that sex has an important effect on aging in certain aspects, and vascular aging also seems to vary by sex [6]. Thus, various vessel-associated diseases, such as stroke, peripheral artery disease, ischemic heart disease, and aneurysms, differ by sex [6]. The existing uncertainty of sex is also likely to affect treatment modalities, and the need for sex-specific treatment options is apparent. Nevertheless, the effect of age, sex, and vessel segment on arterial vasomotor function and gene expression signature has not been explored yet. Thus, we investigated contraction, relaxation, and endothelial integrity, as well as gene expression, in the proximal and distal segments of the thoracic aorta in 6- and 18-month-old mice.

## 2 Materials and methods

### 2.1 Animals

**Fig. 1:**
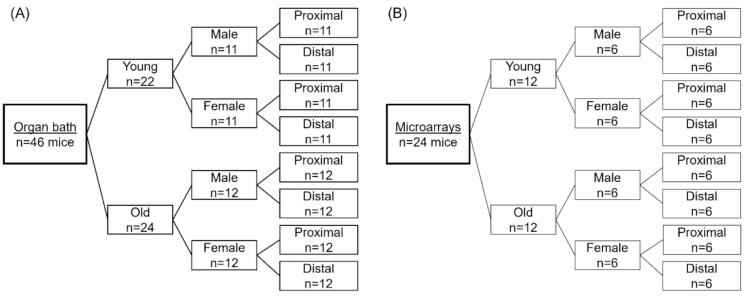
Groups. (A) Functional analysis. (B) Microarrays.

The appropriate Institutional Ethical Committee for Animal Experimentation reviewed and approved this study. We used male and female C57BL/6J mice (Janvier, France) at 6 and 18 months of age for the experiments (Fig. 1). We injected 200 μl (5,7 i.U.) of heparin intraperitoneally. Then, the mice were anesthetized rapidly with isoflurane and euthanized by cervical dislocation.

### 2.2 Harvesting and preparation of the aorta

Immediately after euthanization, the thoracic cavity was opened by bilateral thoracotomy. Then, we carefully exposed and harvested the descending thoracic aorta using microsurgical instruments under microscopic vision. We immersed the harvested aorta in cold carbogenized Krebs-Hensleit solution (KHS) and carefully removed the periadventitial fat and connective tissue under microscopic vision. Then, we cut the harvested aorta into two proximal and two distal rings of 2 mm in length. The proximal and distal segments of the descending aorta were mounted in organ bath chambers for functional investigation. Additionally, the equivalent segments harvested from another mouse of the same sex and age were frozen in liquid nitrogen and stored at −80 °C for molecular analysis.

### 2.3 Organ bath functional experiments

Each aortic ring was mounted in a separate organ bath chamber (EMKA 4 Bath, EMKA Technologies S.A.S, Paris, France) filled with KHS. The KHS was continuously carbogenized and warmed to 37 °C. The rings were initially equilibrated for 20 min at 0.1 g. Then, the tension of the vessel was periodically adjusted to 1.5 g over 60 min, including repeated washing steps with fresh KHS to remove potential metabolites. We exposed the vessel rings to 80 mM potassium chloride (KCl) to test the maximal receptor-independent contractility. When the aortic rings reached a stable plateau, we washed the organ bath chambers with fresh KHS and readjusted the tension to 1.5 g. With a gradually increasing concentration of the α-adrenergic receptor agonist phenylephrine (PE), we induced vasoconstriction, followed by a gradual increase in concentrations of acetylcholine (ACh) to test endothelial-dependent vasorelaxation. To investigate the endothelial-independent relaxation, we induced one further contraction by a single dose of PE (10^−7^) followed by gradually increasing sodium nitroprusside (SNP) concentrations.

### 2.4 RNA preparation

Total RNA was isolated from the descending thoracic aorta's proximal and distal segments. RNA was isolated by TRIzol (Thermo Fisher Scientific, Waltham, USA) extraction. Therefore, the samples were homogenized using a Tissue Lyser II (Quiagen, Netherlands). Then, chloroform was added to induce phase separation. After centrifugation, the upper phase was agitated by incubation with isopropanol. Then, we pelletized the RNA by centrifugation at 4 °C and washed the pellet with NaAc. Then, the pellet was dissolved overnight at −20 °C in DEPC-H_2_O, followed by two washing steps with 80 % ethanol. Then, the RNA was stored in DEPC-H_2_O at −80 °C.

### 2.5 Microarrays

First, we assessed the RNA integrity using the Bioanalyzer (2100 Bioanalyzer, Agilent, USA). We determined the RNA concentration using Nanodrop One (Thermo Fisher, USA). Biotin-labeled ss-cDNA was synthesized from total RNA with a GeneChip™ WT Pico Reagent Kit (Thermo Fisher Scientific, Waltham, USA), fragmented, and subsequently hybridized using mouse arrays (Thermo Fisher Scientific, USA). Afterward, the chips were washed and scanned by the Affymetrix GeneChip Scanner 7G.

### 2.6 Statistics

Vasomotor functional results are expressed as mean ± standard error. We tested for homogeneity of variances by the Levene test using IBM SPSS Statistics for Windows (Version 25.0, IBM Corp., Armonk, NY, USA). Functional data was analyzed using an ANOVA with Tukey adjustment of *p*-values in case of variance homogeneity and Games-Howell adjustment in case of variance inhomogeneity. A value of *p* < 0.05 was considered statistically significant.

The gene expression analysis was performed using R [7]. The gene expression was normalized according to 22 stable mouse housekeeping genes from the House and Reference Transcript Atlas database (Supplemental), selected for consistent expression across conditions [8]. We evaluated the samples by age, sex, and segment using a comparison grid, aligning segment differences with age and sex; same-site comparisons required an additional variable match. The expression differences were analyzed using the Wilcoxon rank-sum test. The *p*-values were adjusted for multiple comparisons using the Benjamini-Hochberg method. Significant and relevant differences were defined as *p* < 0.05 and a minimum 2-fold change in regulation. Genes were prioritized if significant in at least four comparisons to ensure robust differential expression. The pathway enrichment analysis was performed using the WikiPathways 2024 Mouse database to identify significant pathways at adjusted *p* < 0.05. We assessed the age, sex, and segment effects of significant genes using an Aligned Rank Transform (ART) method. Differentially expressed genes were analyzed for co-expression across the conditions. We reported F-values for age, sex, and segment effects for significantly regulated genes (*p* < 0.05 from ART) of each pathway. We conducted pairwise gene correlation analyses using Kendall's coefficient. The correlation matrices evaluated the co-expression across conditions: young versus old, female versus male, and proximal versus distal sites. Only significant correlations (*p* < 0.05) were reported. The heat maps were clustered according to pathways.

## 3 Results

### 3.1 Aortic vasomotor function

#### 3.1.1 Age-related effects

**Fig. 2:**
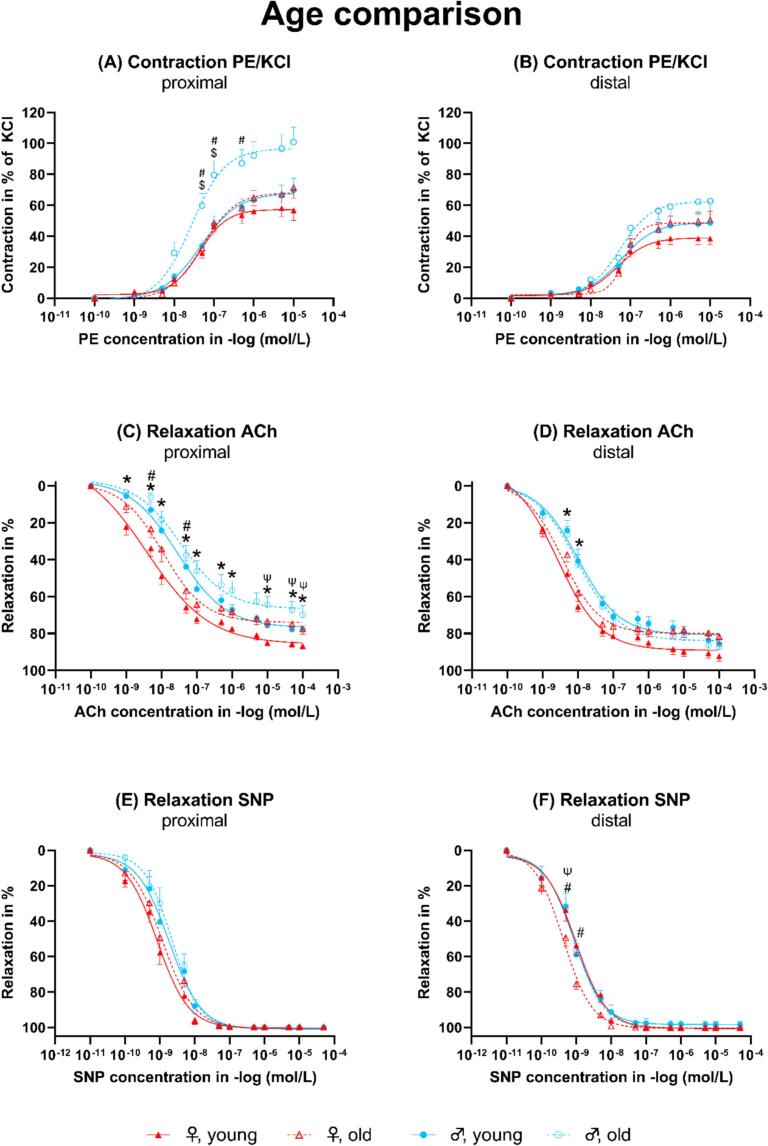
Dose-response curves for the age comparison. ACh: Acetylcholine. PE: Phenylephrine. KCl: Potassium chloride. SNP: Sodium nitroprusside. *Young female vs. young male. ^#^Old female vs. old male. ^$^Old male vs. young male. ^Ψ^Old female vs. young female. All *p* < 0.05.

The contraction to PE in % of KCl was significantly higher in the proximal segment of the descending aorta of old male mice compared to young male and old female mice (Fig. 2A). In the proximal segment of old female mice compared to young female mice, the contraction to PE in % of KCl was not significantly different. In the distal segment, the contraction to PE in % of KCl was only slightly but not significantly higher in old compared to young female or male mice (Fig. 2B). The relaxation to ACh was significantly decreased in the proximal segment of old female mice compared to young female mice (Fig. 2C). In old male mice compared to young male mice, a similar change in age was visible but not statistically significant. In the distal segment, the ACh-mediated relaxation was similar in old males compared to young male mice, but only slightly, and not significantly worse in old females compared to young female mice (Fig. 2D). The SNP-mediated relaxation was comparable between the segments from old and young mice (Fig. 2E). However, in the distal aorta, segments from young female mice relaxed significantly better than those from old female mice (Fig. 2F).

#### 3.1.2 Sex-related effects

**Fig. 3:**
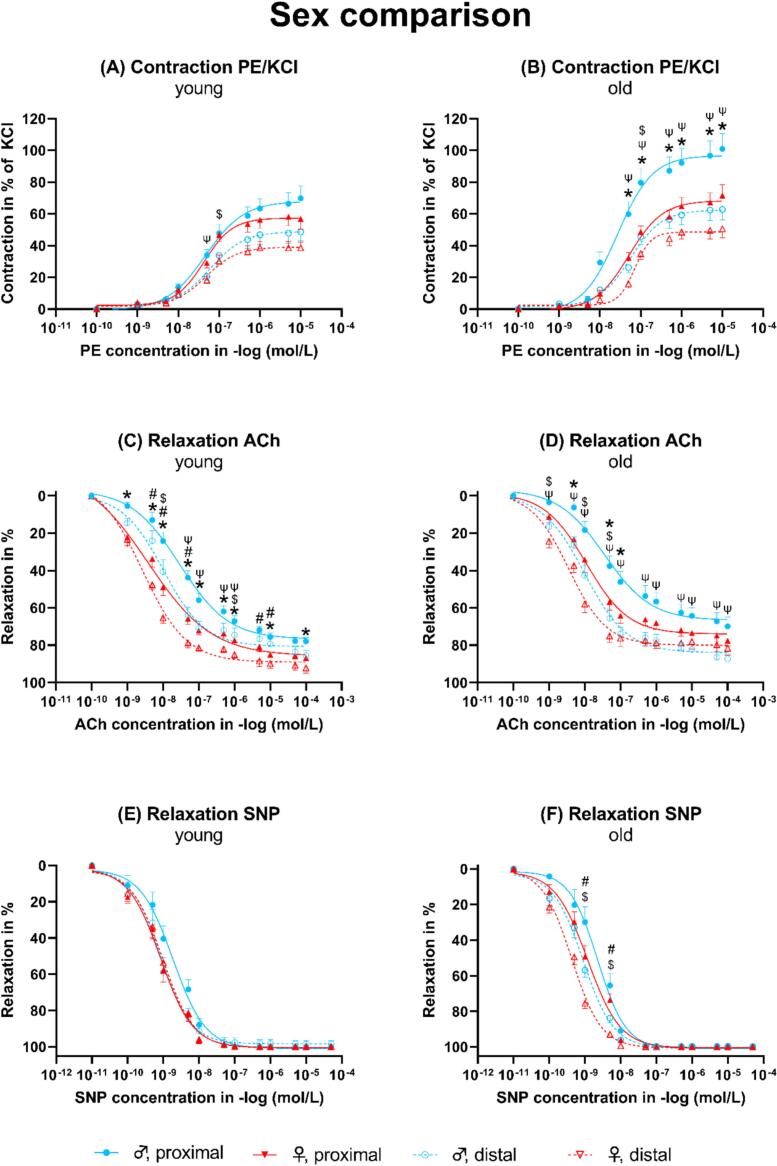
Dose-response curves for the sex comparison. ACh: Acetylcholine. PE: Phenylephrine. KCl: Potassium chloride. SNP: Sodium nitroprusside. *Female proximal vs. male proximal. ^#^Female distal vs. male distal. ^$^Female distal vs. female proximal. ^Ψ^Male distal vs. male proximal. All p < 0.05.

The contraction to PE in % of KCl was slightly but not significantly higher in both the proximal and distal segments of male compared to female mice (Fig. 3A). In old animals, the proximal segment of male mice showed a significantly stronger contraction to PE in % of KCl compared to the proximal segment of female mice (Fig. 3B). The endothelial-dependent relaxation to ACh in young animals was significantly higher in both the proximal and distal segments of female compared to male mice (Fig. 3C and D). In old animals, only in the proximal segment, female mice showed a significantly higher relaxation compared to male mice (Fig. 3D). In young animals, the endothelial-independent relaxation was comparable between both sexes and segments.

#### 3.1.3 Segment-related effects

**Fig. 4:**
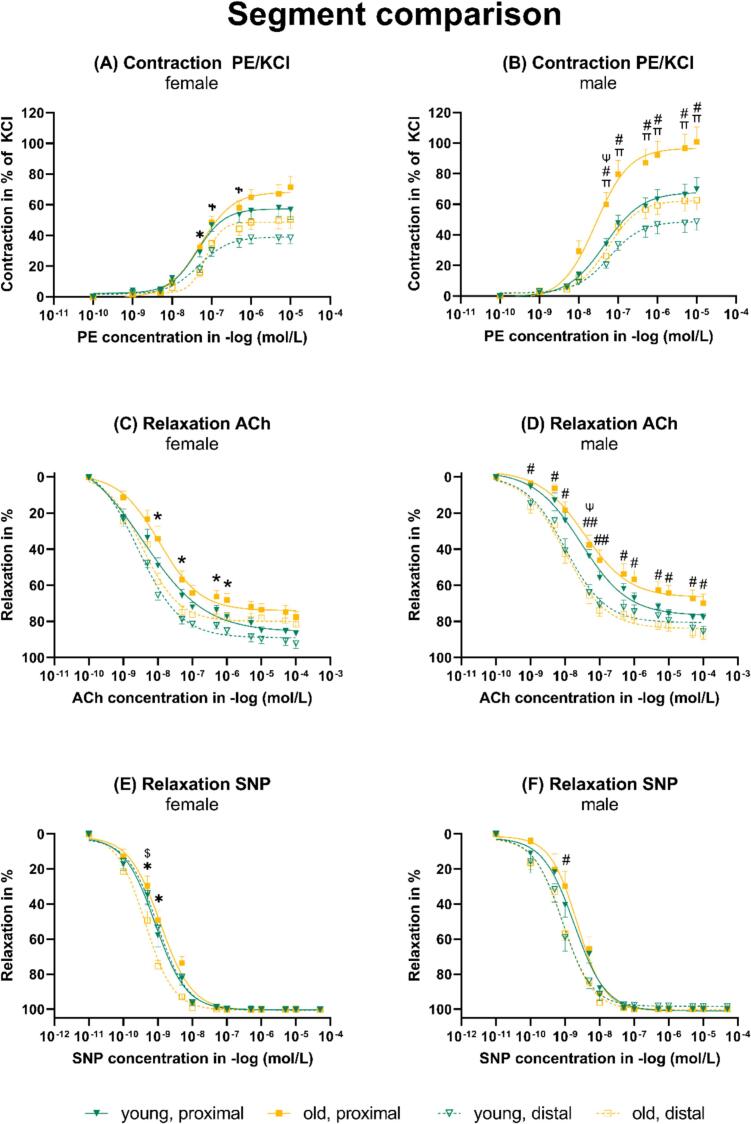
Dose-response curves for the segment comparison. ACh: Acetylcholine. PE: Phenylephrine. KCl: Potassium chloride. SNP: Sodium nitroprusside. *Old female distal vs. old female proximal. ^#^Old male distal vs. old male proximal. ^$^Old female distal vs. young female distal. ^Ψ^Young male distal vs. young male proximal. ^Ⴕ^Young female distal vs. young female proximal. ^π^Old male vs. young male. *^,#, $, Ψ, Ⴕ^P < 0.05. ^##^*P* < 0.001.

In female and male mice, the contraction of PE in % of KCl was significantly higher in the proximal compared to the distal segment of both young and old individuals (Fig. 4A and B). In male mice, the contraction of the proximal segment was higher compared to the distal segment (Fig. 4B). The relaxation to ACh was significantly higher in female and male mice in the proximal compared to the distal segment of old individuals (Fig. 4C and D). In young male mice, the relaxation to ACh was also significantly higher in the distal compared to the proximal segment (Fig. 4D). The relaxation to SNP was also significantly higher in female and male mice in the distal compared to the proximal segment of old individuals (Fig. 4E and F).

### 3.2 Gene expression

**Fig. 5:**
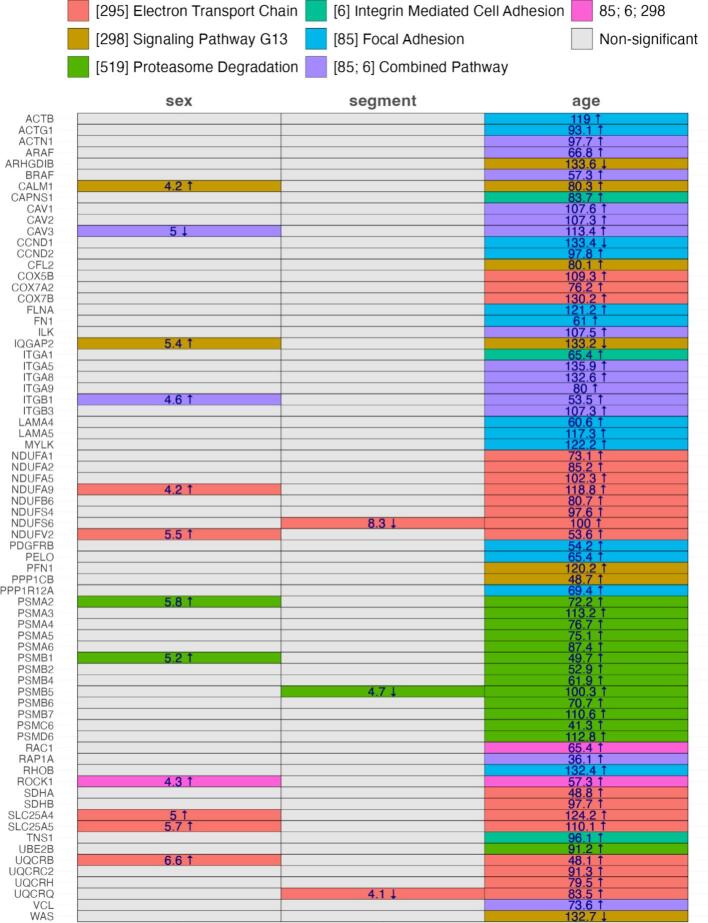
Enriched pathways. Significantly regulated (fold change > or < 2) genes and their pathways are color coded. The numbers represent the F-values of significantly (p < 0.05) enriched pathways. Upward arrows indicate an upregulation in male compared to female, distal compared to proximal, and old compared to young; downward arrows indicate the opposite regulation.

The most significantly enriched pathways, which also have high F-values, appeared in the age comparison (Fig. 5). Some pathways were also enriched in the sex or segment comparison, but no pathway was enriched in both sex and segment comparison, in addition to the age comparison. In the age comparison, genes involved in electron transport chain, such as *COX5B, -7A2, 7B, NDUFA1, -A2, -A5, -A9, —B6, —S4, —S6, V2, SDHA* and *-BSCL25A4* and *-5, UQCRB, —C2, -RH, and -RQ* were expressed lower in old compared to young animals. However, only *NDUFA9* and *—V2, SLC25A4* and *-5,* and *UQCRB* were also enriched in the sex comparison, with a higher expression in male compared to female mice, and only *NDUFS6* and *UQCRQ* were also enriched in the segment comparison, with a lower expression in the distal compared to the proximal segment. Other genes involved in signaling pathway G13, which describes the alpha subunit of heterotrimeric G-proteins, *ARHGDIB, IQGAP2,* and *WAS* were expressed higher levels, and *CALM1, CFL2, PFN1,* and *PPP1CB* were expressed lower in the aorta of old animals. Of those, only *CALM1* and *IQGAP2* were also enriched in the sex comparison and were expressed at higher levels in male compared to female mice. Some enriched pathways were also involved in proteasome degradation, such as *PSMA1* to *6, PSMB1, -2,* and *4* to *7, PSM6C* and *—D6*, and *UBE2B,* in the age comparison, with a lower expression in old compared to young mice. Of those, *PSMA2* and *PSMB1* were enriched in the sex comparison, both with a higher expression in male compared to female mice, and *PSMB5* was enriched in the segment comparison, with a lower expression in the distal compared to the proximal segment. Other pathways were enriched only in the age comparison, such as integrin-mediated cell adhesion, including *CAPNS1, ITGA1*, and *TNS1*, or focal adhesion, including *ACTB,* and *ACTG1, CCND1,* and *CCND 2, FLNA, FN1, LAMA4* and *− 5, MYLK, PDGFRB, PELO, PPP1R12A,* and *RHOB,* all except *CCND2* expressed higher in young compared to old animals. Other regulated genes were involved in both integrin-mediated cell adhesion and focal adhesion, in the age comparison, such as *ACTN1, A-* and *BRAF, CAV1* to *3*, *ILK, ITGA5, −5, −9, ITGB1* and *− 3, RAP1A*, and *VCL,* all with a lower expression in old compared to young mice. Only *CAV3* and *ITGB1*, which were expressed at lower and higher levels, respectively, in male compared to female mice, were also enriched in the sex comparison. As a member of both adhesion types and the signaling pathway G13, *RAC1* was enriched in the age comparison, with a lower expression in old mice, and *ROCK1* was enriched in the age and sex comparison, with a lower expression in old mice and female mice.

### 3.3 Correlations

**Fig. 6:**
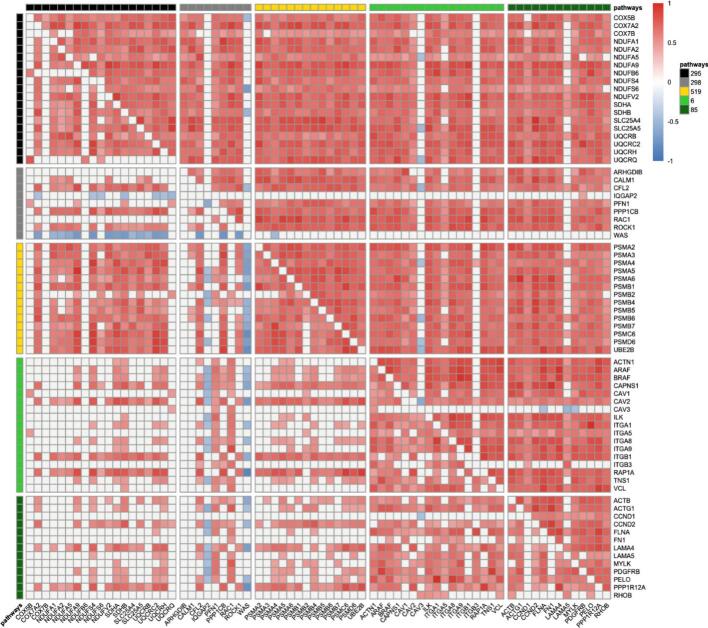
Heat map representing the correlation of genes with each other. The upper triangle represents genes in the aorta of old animals. Male and female as well as proximal and distal segments were combined to generate more stabilization, and only the effect of age was investigated in this analysis. The lower triangle represents genes in the aorta of young animals. Pathway color code: Black (295): Electron Transport Chain. Grey (298): Signaling Pathway G13. Yellow (519): Proteasome Degradation. Light Green (6): Integrin-Mediated Cell Adhesion. Dark Green (85): Focal Adhesion. Correlation color code: Red: positive correlation. Blue: negative correlation. (For interpretation of the references to color in this figure legend, the reader is referred to the web version of this article.)

The heat map (Fig. 6) demonstrates that in young animals, many genes correlate positively within a pathway, except in the signaling pathway G13. Some genes do almost not correlate with any other gene, such as some members of the electron transport chain of the signaling pathway G13. *IQGAP2* and *WAS*, which are involved in the signaling pathway G13, show predominantly negative correlations with the majority of all other genes. In old animals, the majority of genes correlate strongly positively with other genes within the same pathway and with genes of other pathways. Exceptions are *IQGAP2* and *WAS,* which do almost not correlate with other shown genes apart from a few negative correlations with members of the electron transport chain pathway. Other exceptions include CAV3 and ITGB3 from the integrin-mediated cell adhesion pathway and LAMA5 from the focal adhesion pathway, which exhibit only rare correlations with other genes within the same or other enriched pathways.

## 4 Discussion

We investigated the vasomotor function in the proximal and distal segments of the descending thoracic aorta of young and old male and female mice. The response to PE in % of KCl reveals that the endothelial integrity decreases strongly by age in the proximal segment of male mice and only changes minimally in the distal segment. In female mice, it changes only minimally by age in both segments. The response to ACh reveals that the endothelial-dependent relaxation decreases with age in both sexes in the proximal segment and only in female individuals in the descending segment. At a young age, endothelial-independent vasorelaxation is independent of sex and segment, but by age, it becomes superior in the distal segment of female mice. Female sex is associated with high endothelial-dependent relaxation in young age, independent of the segment, and in old age, only in the proximal segment. Endothelial-independent relaxation decreases with age only in the distal segment of female subjects. The less pronounced deterioration of endothelial integrity by age in the segments of female mice could also be associated with the lower prevalence or milder course of distinct cardiovascular disorders in the aged female, such as arteriosclerosis, coronary heart disease, or aortic aneurysms.

The differences in the gene expression signature of the subgroups are diverse and change predominantly with age. The high F-values, visible in the age comparison, also demonstrate that aging has a predominant effect on the regulation of identified relevant genes.

The electron transport chain, which is crucial for energy production in mitochondria, exhibits a decline in function with increasing age [9]. This functional decline is often associated with an increased production of reactive oxygen species [10]. Both a reduced availability of energy and oxidative stress can be reasons for the decreased endothelial-dependent relaxation in old animals. *NDUFS6*, coding for one subunit of the mitochondrial complex I, and *UQCRQ*, both members of the electron transport chain, were expressed even lower in the distal compared to the proximal segment, which might be the reason for worse endothelial-dependent relaxation in the distal segment (Fig. 4C-D). The higher expression of *NDUFA9* and *—V2, SLC25A4* and *− 5,* and *UQCRB* in male mice, which showed lower endothelial-dependent vasorelaxation than female mice, needs more exploration.

The higher contraction to PE in % of KCl indicates reduced endothelial integrity in the aorta of old mice. The G-protein coupled receptor - G13 alpha subunit [11] - signaling pathway, identified in our study, is crucial for the development of the cytoskeleton, endothelial barrier function, and endothelial integrity [12,13]. A recent publication by Liu et al. points out that a reduced *CALM1* expression, which was prevalent in the present study in the aorta of old compared to young mice, seems to lead to reduced endothelial integrity and reduced endothelial nitric oxide synthase function, resulting in a reduced nitric oxide bioavailability, which could be a reason for both, reduced endothelial contraction, and endothelial-dependent relaxation in old, especially male, animals (Fig. 3A-B) [14]. *CFL2, PFN1,* and *PPP1CB* also contribute to maintaining integrity and stability. Consequently, the downregulation of these genes in the aorta from old mice could partially explain the increased contraction to PE in % of KCl. The impact of signaling pathway G13-related genes that were expressed higher in the aorta of old animals on endothelial integrity is unclear based on the current literature: *ARHGDIB* does not seem to have a crucial impact on endothelial integrity [15] *IQGAP2* might be involved in maintaining the endothelial barrier function in the kidney [16]. However, nothing is known about its impact on the vascular endothelium.

Nothing is known about WAS on the vascular endothelium. Nevertheless, Tang et al. showed that the neuronal WAS protein leads to actin polymerization and increased tension in smooth muscle cells and might thereby have an indirect effect on vascular relaxation by inducing stronger contraction beforehand [17]. *CFL2* seems to be more relevant for muscle cells, especially for regeneration and function. Thus, its downregulation in the aorta of old animals might be a reason for the reduced SNP-dependent relaxation (Fig. 2F). However, a direct effect has not yet been assessed.

The proteasome degradation, whose genes were expressed at a lower level in the aorta of old mice, is highly important for the maintenance of the endothelial integrity and endothelial-dependent vasorelaxation, and a reduced proteasome activity is associated with cell senescence and vascular aging [18,19]. The increased expression of the proteasome degradation pathway members, *PSMA2* and *PSMB1,* might also explain the even more pronounced endothelial dysfunction in male compared to female mice (Fig. 3B, D).

Integrin-mediated cell adhesion, whose gene members were underrepresented in the aorta of old mice, is an essential mechanism for the structure and function of the endothelial layer [20,21]. The lower expression of genes associated with focal adhesion in the aorta of old mice might also explain the impaired endothelial function because focal adhesion is crucial for cells to anchor in the extracellular matrix and to allow signal transduction through integrins [22]: e.g., *ACTB* and *-G1* contribute to the maintenance of the shape and barrier function of the endothelial layer [23], *CCND1* promotes endothelial proliferation and survival [24], *FLNA* promotes cell-cell contacts between endothelial cells [25] and *LAMA4* promotes endothelial cell function [26].

*RAC1* promotes eNOS transcription and activity, and its reduced expression might also have contributed to the reduced endothelial-dependent vasorelaxation in the aorta of old mice [27]. The role of *CAPNS1* seems to depend on the type of organ and has not been clarified for the vascular endothelium yet [28,29].

The broad correlation of genes across pathways with each other in the aorta of old animals highlights that the transcriptome is shifted towards endothelial dysfunction, which is not the case in the aorta of young animals. The predominantly negative correlation of *WAS*, which presumably is involved in a higher smooth muscle cell contraction [17], with other genes that are important for the maintenance of endothelial integrity and endothelial-dependent relaxation suggests that the gene interaction promotes a high level of vascular relaxation in the aorta of young animals. Currently, little is known about the effect of IQGAB2 on the vascular endothelium.

### 4.1 Future perspectives

We identified multiple significantly regulated pathways associated with the functional differences of the aorta in dependence on age across both sexes at the gene level. Identified pathways of major interest should be investigated at the protein level in future studies. In case of a high remaining likelihood of impact on vascular function, new treatment options can be developed to target these pathways and counteract the mechanisms and effects of vascular aging. Considering the identified pathways in the present work, potential treatment options are highly diverse. They could reach from a senotherapeutic approach targeting senescence in general, such as senolysis, to distinct molecular targets, including certain inflammatory pathways.

### 4.2 Limitations

Differences between endothelial-dependent and -independent relaxation cannot be explained by gene expression signature in the present study. We did not separately assess the gene expression in the aortic endothelium and smooth muscle cells. Due to the limited amount of available tissue from the murine aorta, we were unable to perform investigations at the protein level.

Aging and its underlying mechanisms can be characterized by twelve hallmarks proposed by López-Otín et al. [30] Those include cellular senescence and a chronic inflammatory phenotype [30]. Whether these hallmarks of aging are expressed differently according to sex and vascular region, and consequently might affect vasomotor function differently, should also be investigated.

## 5 Conclusion

**Fig. 7:**
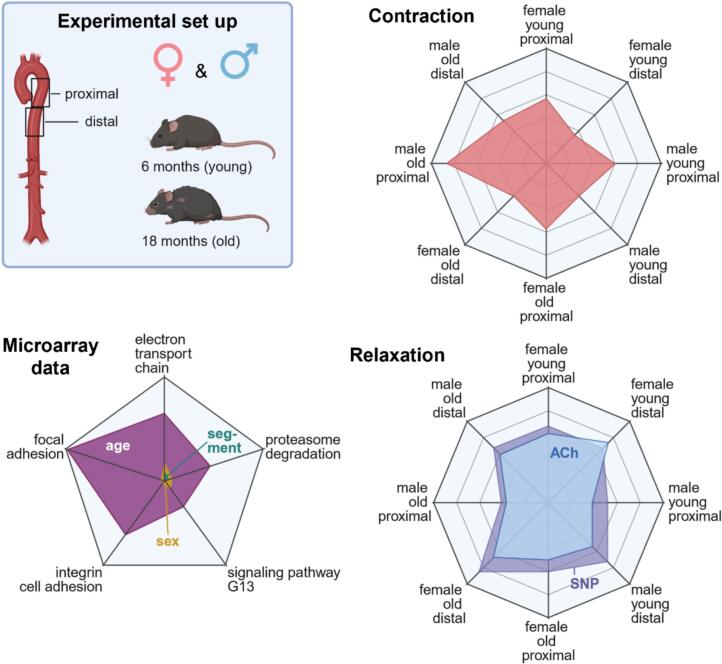
Results overview. ACh: Acetylcholine. SNP: Sodium nitroprusside.

Endothelial integrity and endothelial-dependent relaxation depend on sex and segment of the descending thoracic aorta and deteriorate with age (Fig. 7). The endothelial integrity deteriorates with age, most in the proximal segment of the descending aorta of male mice. Female sex is associated with high endothelial-dependent relaxation at a young age, independent of the segment. However, it decreases with age in both sexes in the proximal descending aorta, only in female mice, and also in the distal segment. The gene expression signature changes markedly with age. The expression of genes associated with endothelial-dependent relaxation, such as members of the electron transport chain, and of genes associated with endothelial integrity and vascular aging, such as members of the signaling pathway of the G13 subunit of the alpha unit of G-proteins, proteasome degradation, and focal or integrin-mediated cell adhesion changes by age, including a few sex-specific differences. The results provide novel insights into vascular aging and may contribute to the development of novel therapeutic strategies to alleviate the pathophysiological effects of vascular aging in humans.

## CRediT authorship contribution statement

**Lars Saemann:** Writing – original draft, Visualization, Supervision, Project administration, Methodology, Investigation, Formal analysis. **Lotta Hartrumpf:** Methodology, Investigation, Formal analysis, Data curation. **Adrian-Iustin Georgevici:** Visualization, Methodology, Data curation. **Sabine Pohl:** Methodology, Investigation. **Anne Großkopf:** Visualization, Conceptualization. **Kristin Wächter:** Methodology. **Yuxing Guo:** Methodology. **Andreas Simm:** Writing – review & editing, Supervision, Formal analysis. **Gábor Szabó:** Writing – review & editing, Supervision, Resources.

## Declaration of Generative AI and AI-assisted technologies in the writing process

The authors did not use generative AI or AI-assisted technologies in the development of this manuscript.

## Funding

This project was supported by the German Research Foundation (10.13039/501100001659DFG; project number 530557324; to Dr. Lars Saemann), by the Wilhelm-Roux funding program of the Medical Faculty of the Martin Luther University Halle-Wittenberg (to Dr. Lars Saemann), and by the 10.13039/501100002347Bundesministerium für Bildung und Forschung (BMBF; project Thera4Age; to Prof. Dr. Andreas Simm).

## Declaration of competing interest

The authors declare the following financial interests/personal relationships which may be considered as potential competing interests: Lars Saemann reports financial support was provided by German Research Foundation. If there are other authors, they declare that they have no known competing financial interests or personal relationships that could have appeared to influence the work reported in this paper.
